# Associations between plasma clozapine/*N*-desmethylclozapine ratio, insulin resistance and cognitive performance in patients with co-morbid obesity and ultra-treatment resistant schizophrenia

**DOI:** 10.1038/s41598-021-81493-0

**Published:** 2021-01-21

**Authors:** Kenya A. Costa-Dookhan, Tarek K. Rajji, Veronica N. Tran, Sylvie Bowden, Daniel J. Mueller, Gary J. Remington, Sri Mahavir Agarwal, Margaret K. Hahn

**Affiliations:** 1 Institute of Medical Science, University of Toronto, Toronto, Canada; 2 Schizophrenia Department, Centre for Addiction and Mental Health, Toronto, Canada; 3 Department of Medicine, University of Toronto, Toronto, Canada; 4 Department of Psychiatry, University of Toronto, Toronto, Canada; 5 Department of Biochemistry and Biomedical Sciences, McMaster University, Hamilton, Canada; 6 Banting and Best Diabetes Center, University of Toronto, Toronto, Canada; 7 Department of Pharmacology and Toxicology, University of Toronto, Toronto, Canada

**Keywords:** Biomarkers, Pharmacology, Endocrine system and metabolic diseases, Schizophrenia

## Abstract

Clozapine (CLZ), the sole antipsychotic with superior efficacy for ultra-treatment resistant schizophrenia (TRS), is limited by adverse effects, including metabolic dysregulation. Clozapine’s main metabolite, *N*-desmethylclozapine (NDMC), has potent 5-HT2C antagonist properties which may explain this metabolic dysfunction, thus the CLZ:NDMC ratio is of particular interest. High insulin resistance states could be associated with CYP1A2 induction and lower CLZ:NDMC ratios. Additionally, lower CLZ:NDMC ratios have been associated with better cognitive, but worse metabolic functioning. This study investigated associations between metabolic and cognitive parameters with the CLZ/NDMC ratio. Primary outcomes included relationships between the CLZ:NDMC ratio to the homeostatic model assessment for insulin resistance (HOMA-IR) and Brief Assessment of Cognition in Schizophrenia (BACS) composite z-scores. Secondary outcomes assessed relationships between CLZ:NDMC ratios to fasting insulin, BMI, weight, fasting glucose, and BACS digit sequencing z-scores. 38 patients who were overweight or obese with schizophrenia or schizoaffective disorder completed fasting bloodwork, anthropometric, psychopathological, and cognitive assessments. Multivariate regressions found a statistically significant inverse association between the CLZ/NDMC ratio and HOMA-IR (B = − 1.028, SE B = .473, β = − 0.348 p = 0.037), which may have been driven by fasting insulin levels (B = − 27.124, SE B = 12.081, β = − 0.351 p = 0.031). The CLZ/NDMC ratio may predict insulin resistance/metabolic comorbidity among patients with TRS receiving clozapine.

## Introduction

Among patients with schizophrenia, 30% have treatment resistant schizophrenia (TRS), which is defined as lack of response to two or more adequate trials of antipsychotics other than clozapine (CLZ)^1^*.* CLZ is the only antipsychotic with an approved clinical indication for TRS, with 40% of treatment refractory patients achieving clinical improvements in symptoms with CLZ treatment^2^. CLZ interacts with dopaminergic, histaminergic, serotonergic, muscarinic, and adrenergic receptors^3^. Despite CLZ’s superior efficacy, 60% of treatment refractory patients will have a poor response^2^ and it carries several serious adverse effects including agranulocytosis, cardiomyopathy, and cardiometabolic dysfunction^4^. CLZ has the greatest metabolic liability of the SGAs^4^, with between 28–45% of CLZ users reported to develop metabolic syndrome over a 4 month or longer period of CLZ treatment^5^.

Both therapeutic and adverse effects of CLZ have been attributed to its main metabolite, *N*-desmethylclozapine (NDMC). CLZ is metabolized to NDMC by hepatic CYP450 enzymes, namely CYP1A2^6^. While NDMC shares some of the same receptor interactions as CLZ, binding affinity at these sites may differ. As example, both NDMC and CLZ are antagonists at 5-HT2 however, 5-HT2C has slightly greater affinity for cortical 5-HT2C receptors^7,8^. 5-HT2C antagonism is linked to weight gain and metabolic disturbances such as glucose dysfunction and insulin resistance, and certain 5-HT2C polymorphisms have been associated with CLZ-induced weight gain^9,10^. NDMC is also a partial agonist at muscarinic M1 receptors, potentially contributing to the pro-cognitive effects of clozapine treatment, and at dopamine D2, and D3 receptors, where NDMC activity may contribute two additional benefits of clozapine treatment: minimal extrapyramidal side effects and improvements in negative symptoms^7,11,12^. Interesting, agonism at these receptor sites has been linked to improvements in certain domains of cognition^13^ and negative symptoms^14^. Therefore, the ratio between CLZ and NDMC (CLZ:NDMC) may have clinical implications regarding both unwanted adverse events and selected clinical benefits. Indeed, previous papers have investigated this relationship, illustrating an inverse relationship between the CLZ:NDMC ratio and cognitive functioning^15–19^, and conversely a positive relationship between the ClZ:NDMC ratio and better cardiometabolic outcomes, including studies examining adjunctive fluvoxamine (a potent CYP1A2 inhibitor) use with clozapine to increase the ratio^20,21^. These results have been summarized in a recent review on the CLZ:NDMC ratio in relation to metabolic indices, cognition, and psychopathology^22^. While these studies illustrate a trend regarding the CLZ:NDMC ratio with respect to these two domains, further research is needed to solidify the relationship.

The primary aims of this cross-sectional study were to investigate the association between the homeostatic model assessment for insulin resistance (HOMA-IR) and cognition (assessed by composite z-scores on the Brief Assessment of Cognition in Schizophrenia (BACS)) in relation to the CLZ:NDMC ratio. In an exploratory investigation, the relationships between the ratio and other metabolic measures, namely fasting glucose levels, body mass index (BMI), and serum insulin levels, as well as the BACS digit sequencing subtest z-scores, were investigated. The BACS digit sequencing subtest is a measure of working memory^23^ and was selected based on a previous study conducted at the Centre for Addiction and Mental Health (CAMH) that reported an association between working memory and CLZ/NDMC ratios^17^.

We hypothesized that lower CLZ:NDMC ratios would be associated with greater insulin resistance (i.e. higher HOMA-IR values), and better cognitive performance (measured by the BACS).

## Experimental procedures

### Participants

We included outpatients patients of both sexes between 17–59 years of age (inclusive) with a diagnosis of ultra-treatment refractory schizophrenia or schizoaffective disorder, defined by having received clozapine treatment for at least 12 weeks at a dose of ≥ 350 mg/day or plasma clozapine levels ≥ 300 ng/mL, a Clinical Global Impression (CGI) Severity score ≥ 4, and/or a score of < 50 on the Global Assessment of Functioning (GAF) scale^24^. In addition, all patients had a BMI > 25. All participants were part of an on-going weight-loss intervention clinical trial (clinicaltrials.gov: NCT02808533), however only baseline measurements prior to randomization of intervention versus placebo were used. This study has been approved by the CAMH Research Ethics Board (REB #097/2015) and all study procedures were completed as outlined in the approved protocol and in accordance with the guidelines and regulations set by the CAMH by the Research Ethics Board. A total of 38 participants underwent informed consent (or informed consent from a parent and/or legal guardian if under 18) approved by the CAMH Research Ethics Board (REB #097/2015) and enrolled in the study.

### Blood sampling/laboratory analysis, anthropometric measures, and cognitive assessments

All 38 participants completed baseline anthropometric measures, medication history (Supplementary Table 1) fasting bloodwork, CGI, and GAF. All assessments were typically done on same day. All cognitive assessments were done under post oral glucose tolerance test, non-fasting conditions to minimize the effect of fasting/non-fasting status on cognitive function. 36 participants completed the Brief Psychiatry Rating Scale (BPRS-Anchored) (18 item) and Positive and Negative Syndrome Scale (PANSS), and 35 participants completed cognitive assessments (i.e. BACS). BPRS-Anchored, PANSS, and BACS were conducted by trained independent raters who are study staff members. Raters had to observe 3 sessions, and were supervised for 3 subsequent sessions (with inter-reliability training), before conducting independent assessments. Fasting serum and plasma samples were collected from all participants, stored at − 80 °C, and analyzed by the CAMH clinical research lab according to their clinical lab reference ranges for plasma indices and drug levels. Fasting insulin was tested on the Beckman Coulter Access 2 autoanalyzer, and fasting glucose with the Siemens Dimension EXL autoanalyzer both following the recommended procedures of the autoanalyzer manufacturing companies, and clozapine levels were measured using a High-Performance Liquid Chromatography (HPLC) method developed in house^25,26^. The HOMA-IR was calculated as follows: HOMA-IR = [Glucose (mmol/L) × Insulin (mU/L)]/22.5. This calculation was derived from fasting glucose and insulin concentrations as a measure of whole body insulin sensitivity^27^. BACS composite z-scores were our primary cognitive measures.

### Statistical analysis

Two multivariable regressions were performed using SPSS version 25 to assess the association between CLZ:NDMC ratios and primary outcomes (HOMA-IR and BACS composite z-scores). All regressions were adjusted for age and gender. Secondary/exploratory analyses were performed with fasting plasma glucose, fasting insulin, BMI, and BACS digit sequencing z-scores as dependent variables.

## Results

### Demographic information

Demographic results are summarized in Table 1. The average age of participants was 39.1 years (± 10.4), majority were male (60.5%), and non-Caucasian (57.9%) The average BMI was 33.8 kg/m^2^ (± 7.40). 78.9% had a diagnosis of schizophrenia. The average CLZ:NDMC ratio was 1.94 (± 0.688) and mean dose of clozapine was 403.9 mg/day (± 160.4).

**Table 1:** Demographic and clinical characteristics of patients.

Characteristic	N	%	M	SD
Age (years)			39.1	10.4
**Gender**				
Male	23	60.5		
Female	15	39.5		
Age of illness onset (years)			23.2	7.57
**Ethnicity**				
Asian	5	13.2		
Black	9	23.7		
Caucasian	16	42.1		
Hispanic	2	5.3		
Mixed	2	5.3		
Indian	2	5.3		
Middle Eastern	2	5.3		
**Diagnosis**				
Schizophrenia	30	78.9		
Schizoaffective Disorder	8	21.1		
**Smoking Status**				
Smoker	16	42.1		
Non-smoker	22	57.9		
Weight (kg)			98.0	23.7
BMI (kg/m^2^)			33.8	7.40
HOMA-IR*			2.95	2.03
Fasting Plasma Blood Glucose (mmol/L)			5.83	0.980
Fasting Serum Insulin (pmol/L)			77.9	53.2
Clozapine dosage (mg/day)			403.9	160.4
Clozapine Level (nmol/L)			2079.9	1072.4
*N*-desmethylclozapine level (nmol/L)			1111.1	576.9
CLZ/NDMC Ratio			1.94	0.688
BPRS*			35.9	11.1
PANSS total*			60.03	19.2
BACS composite z-score**			− 2.23	1.50
BACS digit sequencing z-score**			− 1.29	1.54

*BMI* body mass index, *HOMA-IR* homeostatic model assessment for insulin resistance, *CLZ/NDMC* Clozapine to *N*-desmethylclozapine ratio, *BPRS* Brief Psychiatric Rating Scale, *PANSS* Positive and Negative Syndrome Scale, *BACS* Brief Assessment of Cognition in Schizophrenia.

*N = 36, **N = 35.

### Multivariate linear regression results

**Figure 1:**
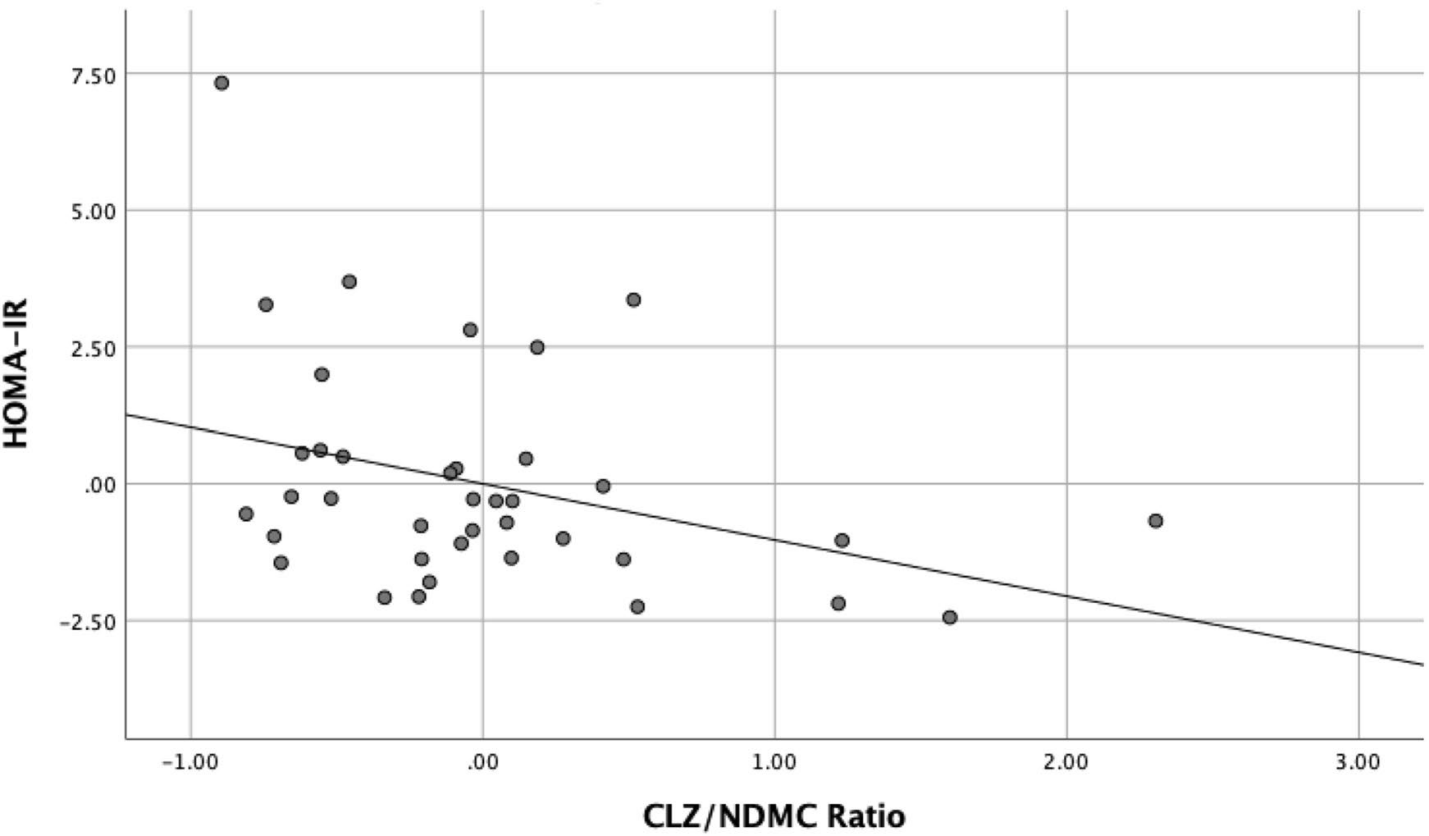
Significant inverse relationship between CLZ:NDMC ratios and HOMA-IR in the study sample. *CLZ:NDMC* clozapine to *N*-desmethylclozapine ratio, *HOMA-IR* homeostatic model assessment for insulin resistance.

**Figure 2:**
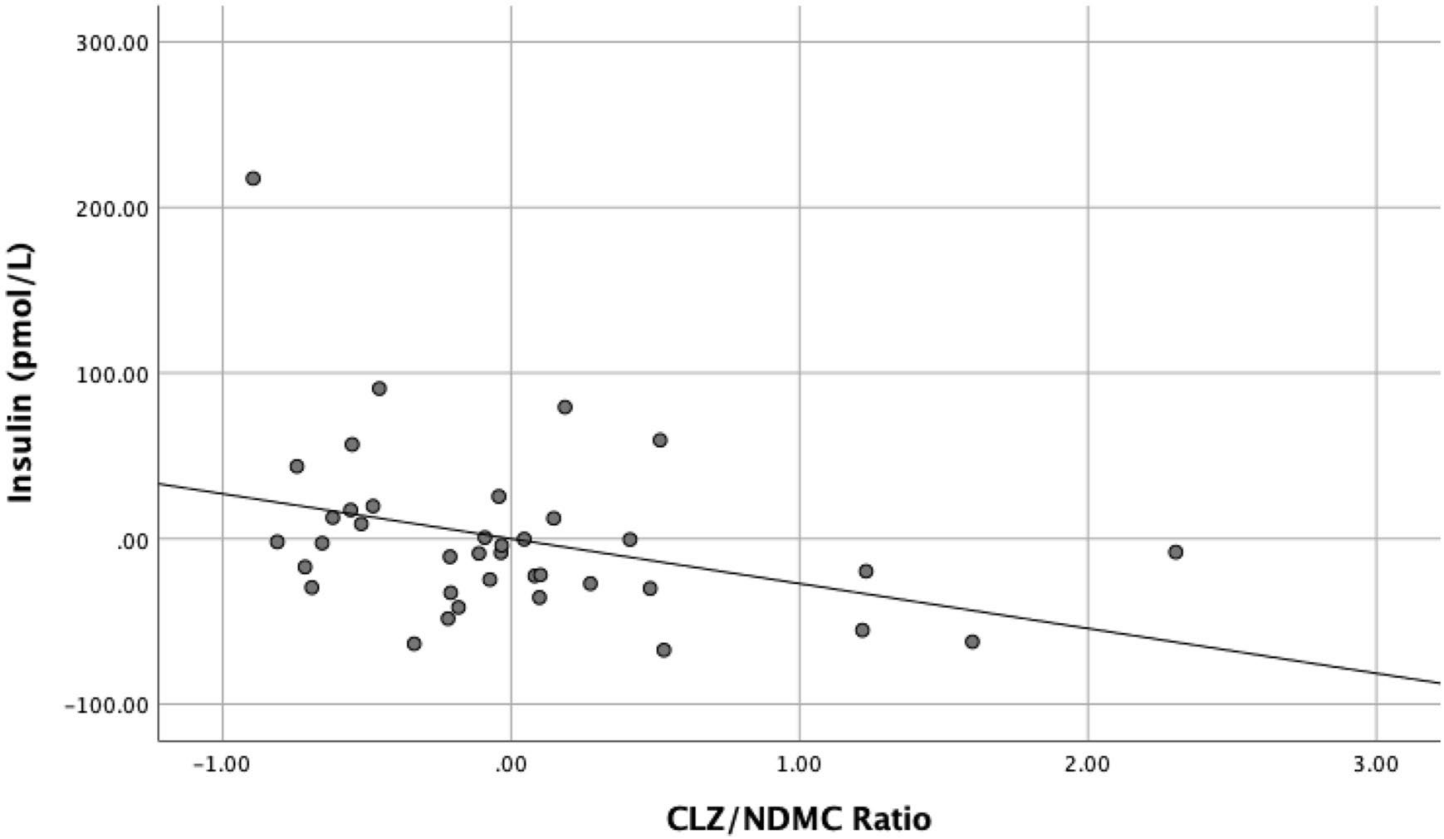
Significant inverse relationship between CLZ:NDMC ratios and insulin in the study sample. *CLZ:NDMC* clozapine to *N*-desmethylclozapine ratio.

As shown in bold in Table 2, a statistically significant inverse association was found between the CLZ:NDMC ratio and HOMA-IR (B = − 1.028, SE B = 0.473, β = − 0.348 p = 0.037) (Fig. 1). Secondary analysis also illustrated a significant inverse relationship between the CLZ:NDMC ratio and fasting serum insulin (B = − 27.124, SE B = 12.081, β = − 0.351 p = 0.031) (Fig. 2). This was not the case for BACS composite z-scores (B = 0.348, SE = 0.346, β = 0.166 p = 0.321) or other exploratory outcomes (Table 2).

**Table 2:** Multivariate regression results for BMI, weight, HOMA-IR, fasting plasma glucose, fasting serum insulin, BACS digit sequences z-scores, BACS composite z-scores.

Dependent variable	B	SE B	*B*	*t*	*p*	95% CI *B*
BMI	− 0.018	1.711	− 0.002	− 0.010	0.992	− 3.50 to 3.46
Weight	− 1.97	5.68	− 0.057	− 0.346	0.731	− 13.52 to 9.58
**HOMA-IR**	− **1.028**	0.473	− 0**.348**	− 2.172	**0.037**	− **1.99 to** − **0.066**
Fasting Plasma Glucose	− 0.155	0.221	− 0.109	− 0.704	0.486	− 0.604 to 0.293
**Fasting Serum Insulin**	− **27.124**	12.081	− **0.351**	− 2.245	**0.031**	− **51.675 to** − **2.573**
BACS composite z-scores**	0.348	0.346	0.166	1.008	0.321	− 0.388 to 0.994
BACS digit sequencing z-score**	0.366	0.387	0.167	0.946	0.352	− 0.423 to 1.156

*BMI* body mass index, *HOMA-IR* homeostatic model assessment for insulin resistance, *BACS* Brief Assessment of Cognition in Schizophrenia.

**N = 35.

## Discussion

Our results show that the CLZ:NDMC ratio is associated with insulin resistance [i.e. a lower ratio or relative increases in NDMC metabolite levels was associated with whole body insulin resistance (Fig. 2)]. These findings complement those of other studies which have found higher CLZ:NDMC ratios to be associated with better metabolic functioning (i.e. as based on BMI, HOMA-IR, fasting serum insulin, and fasting plasma glucose)^20,21,28^. This has also been supported by studies investigating the use of fluvoxamine as an adjunctive treatment for clozapine-treated patients experiencing obesity or other metabolic comorbidities^20,21,28^. In contrast, a recent preclinical study compared acute effects of CLZ as compared to NDMC in relation to glucose metabolism, demonstrating that while NDMC clearly exerted acute metabolic disruptions, these were less pronounced than those induced by the parent compound^29^. The authors hypothesized that this may have been attributable to differential effects of the 2 compounds on insulin responses, which will require further investigation, as well as future studies in humans.

**Figure 3:**
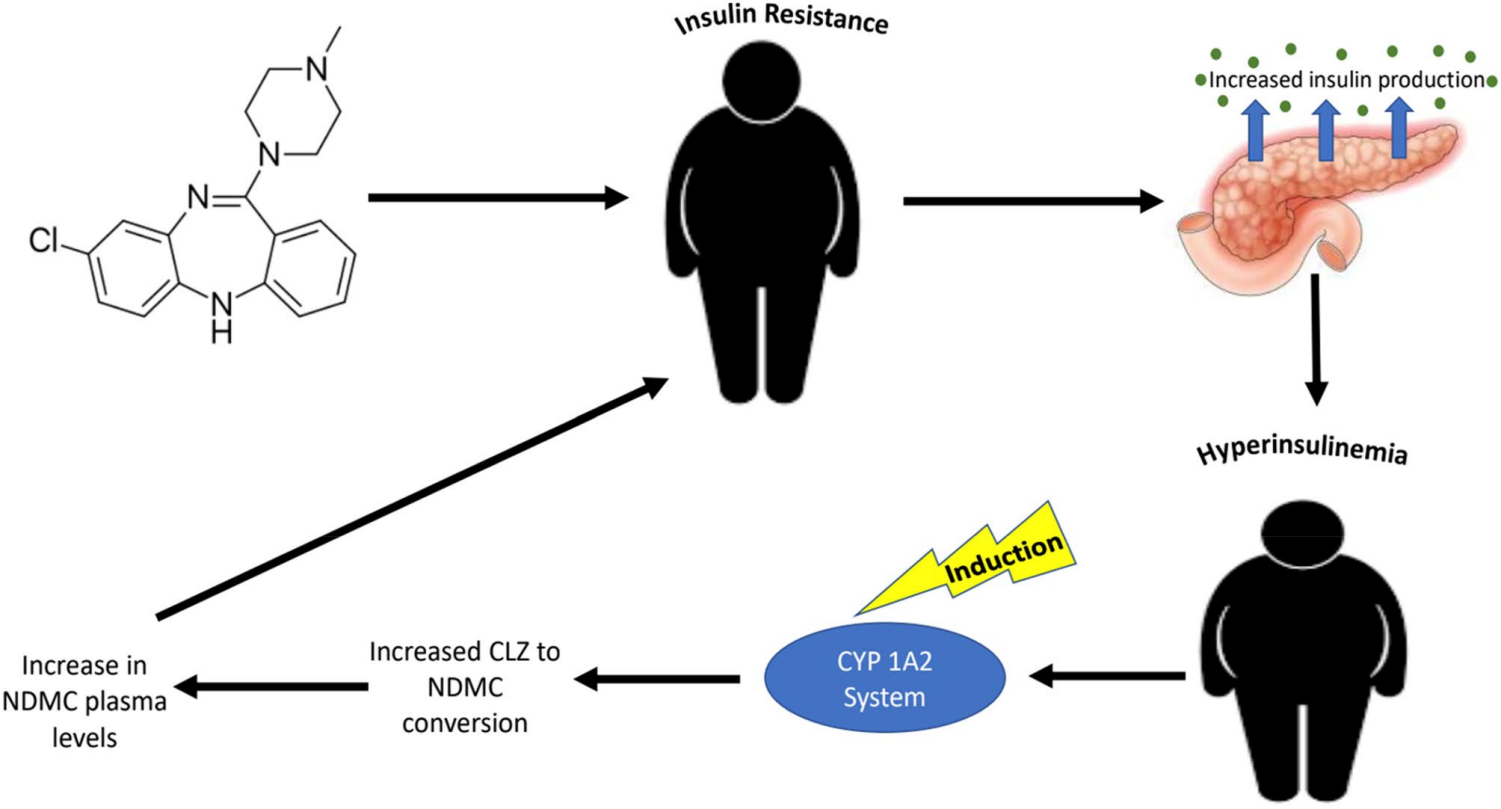
Hypothesized reciprocal association between insulin resistance/HOMA-IR, the CYP1A2 system, and CLZ:NDMC ratio in patients with treatment refractory schizophrenia and obesity receiving clozapine. *CLZ:NDMC* clozapine to *N*-desmethylclozapine ratio, *HOMA-IR* homeostatic model assessment for insulin resistance, *CYP1A2* cytochrome P450 1A2.

In the present clinical study, the absence of a significant association between CLZ:NDMC ratio and BMI may be explained by the limited variability of BMI in our sample, this being attributable to obesity as a study inclusion criterion. The significant inverse association between CLZ:NDMC ratio and HOMA-IR as well as fasting serum insulin levels, but not BMI, could reflect a more complex, reciprocal association between the CYP system and CLZ metabolism (Fig. 3). Interestingly, insulin itself has been reported in a preclinical model to induce the CYP1A2 system^30^, with a parallel literature in humans demonstrating that insulin resistance is accompanied by high circulating peripheral insulin levels. This begs the question of how these findings may translate to patients with insulin resistance. While speculative, it is possible that patients with higher circulating insulin levels, have lower CLZ:NDMC ratios as a result of elevated insulin leading to induction of the CYP1A2 system and increased conversion of CLZ to NDMC. In other words, rather than the CLZ:NDMC ratio being predictive of insulin resistance, insulin resistance and hyperinsulinemia (which are not necessarily synonymous with elevated BMI) may drive the CLZ:NDMC ratios. Larger prospective studies with CYP1A2 genotyping will be necessary to elucidate mechanisms and explain which variables predict the other.

While previous studies have reported CLZ:NDMC ratios to be inversely associated with cognitive functioning, we were unable to replicate these findings^15–19^. Failure to show an association between cognitive functioning and the CLZ:NDMC ratio could be explained by illness severity, as has been proposed previously^31^. For instance, in comparison to the patients in Rajji et al. 2015 study, our patient cohort had higher PANSS scores (M = 60.0, SD = 19.2 compared to M = 53.7, SD = 9.8 for Rajji et al. 2015). This raises the possibility that illness severity may override pro-cognitive effects driven by NDMC. Alternatively, high CLZ levels (reflective of higher CLZ doses used in refractory illness), may saturate the *N*-demethylation process leading to lower conversion of CLZ to NDMC^32^. The lack of association with cognition could also be due to a poorer sensitivity of BACS to working memory dysfunction compared to other cognitive batteries, e.g. MATRICS Consensus Cognitive Battery (MCCB) as used in Rajji et al. 2015. The variability associated with BACS to assess cognitive function in schizophrenia should also be noted as a potential limitation. Despite the observation that Rajji et al. 2015 had a comparable sample size (N = 30), lack of statistical power due to the modest sample size may also be a contributing factor to negative findings. Along with these considerations, use of other medications (i.e. antidepressants, as shown in Supplementary Table 1) may also have influenced cognitive performance and thus the CLZ:NDMC ratio and BACS scores.

Weight gain and metabolic disturbances are among the most prevalent and concerning side effects for patients with TRS receiving CLZ treatment. These adversities negatively affect physical and mental health, medication compliance, and quality of life. The results of this study may contribute to better understanding the mechanisms of CLZ-induced weight gain and support the importance of metabolic monitoring for patients receiving CLZ. Going forward, long-term studies assessing changes in the CLZ:NDMC ratio in relation to severity of illness and metabolic, cognitive, and psychopathological functioning, as well as those that use adjunctive pharmacological interventions to individually target metabolic or cognitive functioning are required. Achieving CLZ efficacy and balancing this with tolerability can be a challenging process for clinicians and patients; therefore, being able to associate the actions of CLZ and NDMC to clinical outcomes and adverse effects early in treatment is critical, and the CLZ:NDMC ratio may present this capability.

## Supplementary Information

Supplementary Table 1.

## Data Availability

All data and materials are available upon request.
