# Supplementary material for: Associations between plasma clozapine/N-desmethylclozapine ratio, insulin resistance and cognitive performance in patients with co-morbid obesity and ultra-treatment resistant schizophrenia
Source: Sci Rep. 2021 Jan 21;11:2004. doi: 10.1038/s41598-021-81493-0 (PMC7820218; doi:10.1038/s41598-021-81493-0)
Supplement: Supplementary file 1 — Supplementary Table 1. [file 41598_2021_81493_MOESM1_ESM.docx]

**Supplementary Information**

**Title:** Associations between plasma clozapine/n-desmethylclozapine ratio, insulin resistance and cognitive performance in patients with co-morbid obesity and ultra-treatment resistant schizophrenia

**Authors:** Kenya A Costa-Dookhan^1,2^, Tarek K Rajji^1,2,4^, Veronica N Tran^5^, Sylvie Bowden^3^, Daniel Mueller ^1,2,4^, Gary Remington ^1,2,4^, Sri Mahavir Agarwal ^$1,2,4^ and Margaret Hahn ^$^*^1,2,4
$^Indicates Shared Senior Authors

*Indicates Corresponding Author

**Affiliations:**

1 Institute of Medical Science, University of Toronto, Canada

2 Schizophrenia Department, Centre for Addiction and Mental Health, Toronto, Canada

3 Department of Medicine, University of Toronto, Toronto, Canada

4 Department of Psychiatry, University of Toronto, Toronto, Canada

5 Department of Biochemistry and Biomedical Sciences, McMaster University, Hamilton, Canada

**Corresponding authors:**

Dr. Margaret Hahn MD, PhD, FRCPC

Centre for Addiction and Mental Health

250 College Street, Rm 707, Toronto, Ontario, M5T 1R8, Canada

Phone: (416) 535-8501 x 34368

Fax: (416) 979-4292

E-mail: [Margaret.Hahn@camh.ca](mailto:Margaret.Hahn@camh.ca)

**Supplementary table 1.** Additional medications received by participants

| Medications | N= |
| --- | --- |
| **Antipsychotic** |  |
| Risperidone | 1 |
| Aripiprazole | 1 |
| **Anti-diabetic** |  |
| Metformin | 14 |
| Sitagliptin | 1 |
| Gliclazide | 1 |
| Glibenclamide | 1 |
| Dapagliflozin | 1 |
| **Anticoagulant** |  |
| Rivaroxaban | 1 |
| **Antiemetic** |  |
| Domperidone | 3 |
| **Beta-blocker** |  |
| Propranolol | 2 |
| Atenolol | 1 |
| Bisoprolol | 1 |
| Nadolol | 1 |
| **Anticholinergic** |  |
| Artane | 1 |
| Benzatropine | 4 |
| Atropine | 5 |
| Oxybutynin | 2 |
| Tiotropium bromide | 1 |
| Ipratropium nasal spray | 1 |
| **Antispasmodics** |  |
| Oxybutynin | 2 |
| **Vitamin and Mineral Supplementation** |  |
| Cholecalciferol | 1 |
| Vitamin B12 | 1 |
| Vitamin D | 2 |
| Vitamin B6 | 1 |
| Ferrous gluconate | 1 |
| Iron | 4 |
| Calcium | 2 |
| Zinc | 1 |
| Euro-fer | 1 |
| **Serotonin–norepinephrine reuptake inhibitor** |  |
| Duloxetine | 1 |
| Venlafaxine | 1 |
| **Benzodiazepine** |  |
| Lorazepam | 5 |
| Clonazepam | 4 |
| **Selective Serotonin Reuptake Inhibitor** |  |
| Citalopram | 2 |
| Escitalopram | 4 |
| Sertraline | 3 |
| **Tricyclic Antidepressants** |  |
| Imipramine | 1 |
| Clomipramine | 1 |
| Amitriptyline | 1 |
| **Norepinephrine-dopamine Reuptake Inhibitor** |  |
| Bupropion | 1 |
| **Triptans** |  |
| Almotriptan | 1 |
| **Proton Pump Inhibitor** |  |
| Rabeprazole | 2 |
| Teva-lansoprazole | 1 |
| Rabiprazole | 1 |
| Pantoprazole | 2 |
| Teva-Pantoprazole Magnesium | 1 |
| **Statins** |  |
| Atorvastatin | 4 |
| Rosuvastatin | 3 |
| **Non-statin Cholesterol Absorption Inhibitor** |  |
| Ezetimibe | 1 |
| **Laxative/Stool Softener** |  |
| Senna glycoside | 7 |
| Lactulose | 2 |
| Polyethylene Glycol | 2 |
| Bisacodyl | 2 |
| Docusate | 7 |
| **Beta-3 Adrenergic Agonists** |  |
| Mirabegron | 1 |
| **Antihypotensive** |  |
| Midodrine | 1 |
| **ACE Inhibitor** |  |
| Enalapril Maleate | 1 |
| Ramipril | 1 |
| **Calcium Channel Blocker** |  |
| Amlodipine | 1 |
| **Nicotine Replacement Therapy** | 2 |
| **Sedative-hypnotics** |  |
| Zopiclone | 2 |
| **Analgesics** |  |
| Acetylsalicylic Acid | 1 |
| Acetaminophen | 1 |
| **Fibrate** |  |
| Gemfibrozil | 1 |
| **Histamine H2-receptor Antagonist** |  |
| Ranitidine | 2 |
| **Anticonvulsant** |  |
| Divalproex | 1 |
| Gabapentin | 2 |
| **Opioid** |  |
| Percocet | 1 |
| **Antiasthmatic** |  |
| Salbutamol | 1 |
| **Bronchodilator** |  |
| Ipratropium | 1 |
| **Corticosteroids** |  |
| Fluticasone | 1 |
| **Antiparkinsonian Agent—Dopamine Agonist** |  |
| Pramipexole | 1 |
| **Antiepileptic** |  |
| Lamotrigine | 1 |
| **Antibiotic** |  |
| Auro-amoxicillin | 1 |
| Isoniazid | 1 |
| **Antimicrobial/Antiviral** |  |
| Glycerin | 1 |
| **Central Nervous System Stimulant** |  |
| Modafinil | 1 |
| **Growth Hormone-inhibiting Hormone** |  |
| Somatostatin | 1 |
| **Antiestrogens** |  |
| Tamoxifen | 1 |
| **Thyroid Stimulating Hormone Medication** |  |
| Levothyroxine | 2 |
